# Monthly Summary

**Published:** 1878-03

**Authors:** 


### MONTHLY SUMMARY.

---

*Unusual Action of Anæsthetics.*—Dr. P. Keyser, in the *Medical and Surgical Reporter*, relates the following cases;

While I was attending Prof. von Graefe's clinic, in Berlin, a young lady of about 19 years came in, for the operation of neurotomy of the supra-orbital nerve. It was necessary to anæsthetize her chloroform being used. During which act, in the presence of the class, she passed through all the actions and emotions of coition. Dr. von Graefe called attention to this, as one of the effects, at times, of inhaling chloroform, and advised the necessity of having several present when chloroform was administered to the opposite sex.

In my clinic in the Willis Eye Hospital, in 1874, a female of seventeen years of age came to me to be operated on for strasmus convergence. She wished to be etherized, so as to prevent her feeling any pain. During the administration of the anæsthetic by the house surgeon, in the presence of several visiting students and physicians, as well as the regular clinic assistants, she, after a few minutes inhalation, began talking in the most lascivious manner, accompanied with all the actions and emotions of sexual intercourse ; and when coming out from under the deep anæsthesia, after the operation, the same hallucination and actions possessed her. When free from its influence she claimed that one of the assistants had had sexual intercourse with her. This idea was given up, however, after being assured that such was not the case, and of the number present at the operation. Some one must have mentioned the whole or part of the affair to her, for on her appearance at the next clinic day she very modestly apologized to me for her actions, saying, " that she could not help it, as she knew nothing at the time."

In the spring of 1876 I was obliged to etherize a young lady, so that I could make the operation of tearing loose posterior synechia, and during the inhalation, which was administered by my friend, Dr. Frank Fisher, the eyes became uncovered for a moment, in which time she opened them and looked staringly and frightened at me, as I sat by her side holding the pulse. She struggled to get away, and attempted to scream. Her eyes were at once covered, and the ether pushed to complete anæsthesia. After coming out from under its influence, she told the young lady friend with whom she was staying, and who was present at the operation, that " I had turned into a wild cat; she was positive of it " For ten days she was so impressed with this delusion that she would not look at me, and it was with difficulty that I could treat the eye."

---

*Chloral for Removing Warts.*—A solution containing about twenty grains of chloral hydrate to the ounce of water, is recommended by Dr. Craig as being effectual for the removal of warts. The operation is said to be painless.—*Canada Med. Record.*

---

*The Liquefaction of Gases.*—It is announced that the successful liquefaction of oxygen, which was effected at Geneva a month ago, by M. Raoul Pictet, has been almost immediately followed by the liquefaction of hydrogen and nitrogen gas and of common atmospheric air. This, it is said, was effected by M. Cailletet, in the presence of three members of the Institute of France, on the last day of 1877. Thus, no gas remains which cannot be condensed into the liquid state under an adequate pressure and with sufficient arrangements for the lowering of the temperature.—*Med. and Surg. Reporter.*

*Supra-Orbital Neuralgia.—(Dr. C. Rœse, Leipsig,* 1876, *Inaug. Dissert.*)—Says the most frequent causes of supra orbital neuralgia are malaria, syphilis, hysteria, cold and injury. Furthermore it accompanies different forms of eye affections, and in some rare cases is due to thickening of the bones of the skull, &c. Typical malarial neuralgia occurs in the young and middle aged, and men are oftener affected than women. Shoemakers, on account of their work, are peculiarly susceptible to neuralgia, but not the typical form. Typical supra-orbital neuralgia is observed in the course of cold or hysteria. The pain of supra-orbital neuralgia generally begins in the morning and lasts from half an hour to six hours. The quotidian type is exceptional. The right supra-orbital nerve is more often affected than the left, and both are very seldom affected together. The proportion of these kinds in 61 cases was 35: 18: 8. In occasional cases a glaucomatous condition and anæsthesia of the retina have been observed. The best remedies in typical cases of neuralgia are quinine and arsenic. Iron is also very useful. Among the preparations large doses of ferrum oxydatum S. hydricum have been well thought of. Hot foot baths are also beneficial, especially when the neuralgia is double. Blistering the forehead or painting it with iodine may also be tried.—*Schmidt's Jahrbucher.—Canada Medical and Surgical Journal.*

---

*General Nervousness in the Male.*—In a lecture reported in the *Medical News and Library*, S. Weir Mitchel, M. D., says: This condition is rarely mentioned in the books. It is not raised to the dignity of a disease, but sometimes it seems necessary to treat it as such: Sometimes it is ephemeral; often it is too lasting. Occasionally it is due to original defects arising at birth, and is carried to the grave. The most prominent peculiarity noticeable in this condition of general nervousness is over-excitability. A state of feeble or tardy nutrition accompanies the nervous condition. The causes are various. Among them may be mentioned enfeebling wounds, general sclerosis, exhausting fever and diarrhœa, paralysis agitans, disorders of the abdominal viscera, self-abuse, sexual excesses, and the use of tobacco.

The treatment resolves itself into a treatment of anæmia, of defects of nutrition aud assimilation, and dyspepsia; also, in a change in the daily habits of life, from indoor pursuit to outdoor life; also, into the use of drugs with moderation. Digitalis for palpitations, opium and cannibais indica are indicated in small doses where they are tolerated. Arsenic for a nerve tonic must be long continued. The bromides are useful, especially the bromide of lithia. The combination of the valerianates of quinia, zinc and strychnia is also good.—*Detroit Lancet.*

*Death from Chloroform Averted by the Inhalation of Nitrite of Amyl.—British Medical Journal* reports a case in which death from chloroform was averted by the timely inhalation of nitrite of amyl. About two drachms had been used, when the patient made an abortive attempt to vomit, raised her head from the pillow, gave a gasp, when the pulse stopped: foam gathered on her lips; her jaw became rigid, and to all appearance she was dead. Her tongue was pulled forward, artificial respiration was resorted to, cold water dashed in her face, but without success. Some nitrite of amyl was poured upon lint, and, as near as could be judged, in about ten seconds there was a flushing of the face, the pulse was again felt, and respiration was restored.—*Dental Lancet.*

---

*Subcutaneous Injections of Chloroform.*—In a note addressed to the Societe de Therapeutique (*Gaz. des Hop.*., Dec. 14,) M. E. Besnier details the results of numerous trials which he has made at the St. Louis, of the hypodermic injection of chloroform, first practiced by Dr. Roberts Bartholow, in 1874. There has been, he observed, but little published upon the subject, probably because these injections have been used only for a limited purpose (the treatment of neuralgia,) instead of employing them for the relief of every kind of pain. Their great advantage, he considers, consists in the fact that they may be employed in this manner, and thus supersede morphia, with its consequent inconveniences. No ill effect whatever, local or general, follows these injections, and yet they are efficacious. But the mode in which they are made is of importance, for, inefficiently performed (which is very commonly the case,) they may give rise to local phlegmasia. They should always be practiced in two stages, the needle being first separated and introduced, alone, so that if it happen to penetrate a vein this may be made known by the issue of a droplet of blood. When the syringe has been reapplied, in order to prevent local irritation being caused, the injection should be propelled into the hypodermis (*i. e.*, the subcutaneous cellulo-adipose layer, which varies in thickness in different regions and individuals,) which not only possesses a special tolerance and insensibility, but a very active absorbent power. If the point of the needle be very fine and sharp, it may be passed through the skin into this tissue without appreciable pain, which, however, will be felt if it be carried too far, so as to reach the muscles, etc. The needle is manœuvred with the greatest facility as soon as it has passed the dermis, its point being easily guided to any part of the hypoderm. This done, the syrine may be adapted, and the injection made, with the greatest security.—*Med. and Surg. Reporter.*

*Can Twenty Grains of Chloral Kill?*—This valuable medicine, like most useful medicines, is not without its dangers and drawbacks. Occasionally is recorded an account of death supposed to have been caused by an overdose. What is a proper and safe quantity to be administered at one time? A drachm would hardly be thought to be dangerous. Less than this, however, is usually enough to promote sleep. Dr. Ingalls in the Chicago *Med. Journal* gives an account of the case of a healthy woman to whom 20 grains of chloral in two doses, with an interval of an hour, were given with the intent of preventing pain in the extraction of teeth. She showed symptoms of poisoning soon after the administration of the second quantity, and in spite of remedies was dead in fifteen minutes! Why did that woman die? Could twenty grains of chloral kill? It may be doubted. But it will be well not to forget such facts as this, and always maintain due caution in prescribing a remedy of which such a result is maintained to be possible. Liebreich says that only the crystallized drug is safe. The impure preparation poisons, sometimes, and even may kill.—*Druggists' Circular.*

---

*The Odors of Persons.*—A curious contribution to neurology is contained in an article by Dr. William A. Hammond, reprinted from the *Transactions* of the American Neurological Association. It is descriptive of the peculiar odors given off by the human body in certain conditions and affections of the nervous system. Thus he tells of a young married lady, of hysterical tendencies, who, during her paroxysms, exhaled an odor of violets, which must have in some measure reconciled her husband to these unpleasant domestic occurrences. Another lady, the doctor reports on strong testimony, "during the venereal excitement, gives off a very rosaceous odor." But the peculiarity is not always thus charming. A young lady, a school teacher, subject to sick headaches, evolves at these periods, *horrible dictu*, "an odor similar to that of Limburger cheese!" Then there is the "odor of sanctity," which Dr. Hammond also touches upon, and which he does *not* identify necessarily with the agreeable ones. His study is of the most curious.—*Med. and Surg. Reporter.*

---

*Deaths from Anæsthetics in* 1877.—We have recorded in our columns, during the present year, fourteen cases of deaths under anæsthetics. Ten of these deaths were from chloroform, one from ether and nitrous oxide gas, one from methylene and ether, one from nitrous oxide gas, and one from ether and carbonic acid gas. The patient in the latter case (a man sixty-nine years of age, suffering from peritonitis,) having for about thirty seconds inspired only his expired air.—*New York Medical Jour.*

*Circassian Surgery.*—An English surgeon in the Turkish army writes this incident illustrating surgical knowledge among the Circassians :—"On passing the Circassian camp on my way home, I was hailed by an orderly of the Pacha, to know if I would kindly call and see a wounded officer. Of course I went, and found, among several others wounded, a very handsome young officer, with a bullet somewhere in the elbow. Attending upon him, I found a wild-looking old Circassian, who was sucking the wound, in the vain hope of thus extracting the bullet, the simplest and safest way, he assured me, of performing this operation. After this interesting experiment had been continued until the operator was thoroughly exhausted, and without, I regret to say, his labors being crowned with success, the mollah or priest, was called in consultation ; but by this time the Pacha, who was present, and who wished me to think him superior to the superstitions of his people, asked me to interfere. I did so, on the condition that I should have the officer removed to our ambulance, which that morning had come into existence, though in a very small way, it is true. To this he consented ; and, after his arrival, I put him under chloroform, and found that a good deal of sucking or enchantment would have been required to extract the ball, which had entered the ulna, and carried before it a portion of the olecranon, which was wedged firmly, together with the bullet and fragments of clothing, between the split condyles of the humerus, right into the cancellous tissue. Having cleared out all the fragments of bone, I found that a sufficiently complete "resection" of the joint had been made without my further interference; and, under the nearest approach to antiseptic measures which I have at command, in this fresh healthy air, and with the robust constitution of the patient, I hope to save him a tolerably useful arm.—*Med. and Surg. Reporter.*

---

*Arsenic and Antimony in Vulcanized Rubber.*—An article contained in No. 17 of the *Repertoire de Pharmacie*, by Mr. E. Filhol, again draws attention to the fact that much of the vulcanized rubber of the market, at least that made in Europe, contains arsenic, which may be readily extracted by means of hydrochloric acid. It may not be generally known that the golden sulphide of antimony is largely consumed for coloring vulcanized rubber. This is prepared specially for the purpose, both of a red and of an orange color, and preserves its tint even at a high temperature. Some time ago an inquiry was instituted whether dental vulcanite, colored by vermilion, was poisonous ; and the result was that it was declared innocuous. It now remains to be seen whether any dental vulcanite is colored by antimony sulphide, and what constitutional effects it produces.—*Med. and Surg. Reporter.*

*Chloroform Hallucination and Alleged Rape.*—A surgeon was recently indicted at the Northampton Assizes (England) for rape, while having a tooth extracted. The offence charged was alleged by the prosecution to have been committed while tne prosecutrix was so far under the effect of chloroform or chloric ether as to be speechless and motionless, but not unconscious. The defence set up was that the prosecutrix was under the influence of chloroform to such an extent as to have caused her to imagine that to have been done which she described. For the defence, Dr. B W. Richardson was called, and stated from the evidence in the present case he should say that the patient had reached the second stage. The usual symptoms accompanying that stage were loss of consciousness and, in the case of women, development of any hysterical tendencies, any operation being impossible at this stage, the patient resisting and screaming if touched. It was at this period that the patient was subject to illusions. A person who was deprived of the power of motion by chloroform would, in his judgment, be deprived of the power of sight. He knew from his own personal experience that persons in the second stage were subject to delusions as to what had been done to them during the time. He gave an instance of a lady who, in the presence of himself, her father and mother, and a dentist's assistant, while under the influence of chloroform, brought a charge against the dentist who was operating upon her precisely similar to the one in the present case, and continued firm in the belief that the charge was well founded long after the influence of the chloroform had passed off, and probably still continued in that belief. The other medical witnesses who were called, and who expressed their concurrence in Dr. Richardson's evidence, were Mr. Mills, administrator of chloroform at St. Bartholomew's, Dr. Hawksley, Dr. Saundly, of Birmingham, and Mr. West, Senior Surgeon of Queen's College Hospital, Birmingham. It was stated that all the medical witnesses for the defence had come forward to give their evidence entirely gratuitously. The jury rendered a verdict of *Not Guilty.*

*Moral.*—Medical gentlemen should never administer anæsthetics to a female unless in the presence of witnesses.—*Med. Record.*

---

*Iron Cement.*—Powdered sal ammoniac, 2 ounces. Flowers of sulphur, 1 ounce. Iron filings, 5 pounds. Water sufficient.

Used for closing the joints of iron pipes, iron and glass skylights, etc. No more of the cement should be made at a time than can be used at once.—*The Pharmacist.*

